# Supported Zeolite Beta Layers via an Organic Template-Free Preparation Route

**DOI:** 10.3390/molecules23010220

**Published:** 2018-01-21

**Authors:** Stephanie Reuss, Dirk Sanwald, Marion Schülein, Wilhelm Schwieger, Shaeel A. Al-Thabaiti, Mohamed Mokhtar, Sulaiman N. Basahel

**Affiliations:** 1 Department of Chemical Reaction Engineering, Friedrich-Alexander Universität Erlangen-Nürnberg, Egerlandstraße 3, 91058 Erlangen, Germany; stephanie.reuss@fau.de (S.R.); dirk.luedke@fau.de (D.S.); marion.schuelein@fau.de (M.S.); 2 Chemistry Department, Faculty of Science, King Abdulaziz University, 21589 Jeddah, Saudi Arabia; sthabaiti@kau.edu.sa (S.A.A.-T.); mmoustafa@kau.edu.sa (M.M.); sbasahel@kau.edu.sa (S.N.B.)

**Keywords:** zeolite layer, zeolite beta, organic structure directing agent free, coatings, membranes

## Abstract

Layers of high silica zeolites, synthesized with an organic structure directing agent (OSDA) and grown onto porous support structures, frequently suffer from the thermal stress during the removal of OSDA via the calcination process. The different thermal expansion coefficients of the zeolite and the support material, especially when stainless steel is used as a support, causes enormous tension resulting in defect formation in the zeolite layer. However, the calcination is an easy procedure to decompose the OSDA in the pore system of the zeolite. Recently, methods to synthesize zeolite beta without the use of an organic structure directing agent have been described. In the present study, a seed-directed synthesis is used to prepare OSDA-free zeolite beta layers on stainless steel supports via an in situ preparation route. For the application as membrane, a porous stainless steel support has been chosen. The beta/stainless steel composites are characterized by X-ray diffraction (XRD) and scanning electron microscopy (SEM). To prove its possible application as a membrane, the beta/stainless steel composites were also tested by single gas permeances of H_2_, He, CO_2_, N_2_, and CH_4_.

## 1. Introduction

The past two decades have seen tremendous advances in the areas of the preparation, microstructure characterization, permeation mechanism and application of zeolite-based layers and coatings. It is generally accepted that the zeolite membranes offer superior thermal, chemical and mechanical stability in harsh environments, in contrast to the polymeric membranes. Due to the large diversity of available zeolite materials, support structures and the tunability, the zeolite properties utilizing various numbers of post-treatment methods, many applications for zeolite coatings and membranes have been achieved [1,2,3,4,5]. However, just a few applications have been realized on a technical or pilot scale level. Interesting applications are, most notably, separation tasks [6] and sensor applications [7], as well as the use as a catalytic membrane reactor [8]. Especially for the application as a membrane either for separation or as a catalytic membrane, dense and defect-free layers are necessary. It is critical to prevent defects or voids that might exist in addition to the desired zeolite pores to improve the potential of zeolite coatings for further industrial applications. Such voids and defects reduce the membrane properties to separate molecules based on their size. The formation of such defect might be because zeolite membrane layers are intergrown polycrystalline assembles with the grain boundaries invariably existing between crystals or crystallites. In addition, such intercrystalline voids are generated by thermal treatment or calcination processes, which are necessary to remove the organic structure directing agent (OSDA) from the zeolite’s pore system. Even if slow heating and cooling rates are applied during the calcination process, the formation of defects can occur. To avoid this problem, several groups, in recent years, have studied OSDA-free synthesis methods for MFI-type zeolite layers and membranes where an organic structure directing agent is usually necessary [9,10,11,12,13,14,15]. Most of them performed an in situ or ex situ seeding with silicalite-1 crystals followed by a secondary growth step. Okubo et al. [16] show a detailed overview for the OSDA-free synthesis of some zeolites with a special focus on zeolite beta.

Not only MFI zeolite membranes and layers have shown great potential as catalytic coatings and membranes, but zeolite beta has also attracted more attention in recent years [17,18,19,20,21,22]. Zeolite beta is a high-silica zeolite, which can be crystallized on various supports in the form of very well intergrown layers. In addition, zeolite beta membranes show a good performance for organic vapor separations and as membrane reactors due to their specific pore width and a three-dimensional pore system with channel-like geometries. Until now, they have been prepared using an OSDA via seeding and secondary growth methods [19,20,21] or via direct synthesis methods [17,18,22]. 

The group of Xiao [23] reported one of the earliest attempts on seed-induced crystallisation of zeolite beta powder in the absence of an OSDA. The synthesis recipes were further improved by Xiao’s group [24,25,26,27] themselves and by the groups of Okubo [16,28,29,30,31] and Mintova [32]. In these reports, calcined and uncalcined zeolite beta seed particles, produced earlier with an OSDA, are used in a secondary growth synthesis solution where indeed no additional OSDA is required. The reported synthesis procedures vary in terms of the kind and amount of seeding particles, the content of the secondary growth solution, in synthesis times and temperatures. The most recent publications favor lower synthesis temperatures around 120 °C, which then requires a longer synthesis time up to 120 h to reach high crystallinities and yields [24,25,26]. Yilmaz et al. [25] have also pointed out economic advantages of OSDA-free zeolite beta as compared to conventionally prepared zeolite beta powder. Thus, under economic aspects, the costs that can be saved by the use of a minimum amount of OSDA and even the effort to avoid the calcination step at high temperatures are an advantageous aspect of an OSDA-free beta synthesis. This is especially the case when zeolite beta that is already OSDA-free can be used as seed material, which Okubo et al. investigated [30]. The published reports show that, in general, OSDA-free prepared zeolite beta exhibits a larger particle size, lower Si/Al ratio (4.5 to 5) and larger micropore surface area. Crystallinity is also higher due to a smaller number of structural defects in the crystal lattice [25]. 

The group of Tang et al. [33] described recently the OSDA-free synthesis of beta zeolite membranes on porous α-Al_2_O_3_ support. Still, calcined beta seeds here, which originally were synthesized with an OSDA, were ex situ deposited via spin-coating on a porous alumina support, followed by an OSDA-free secondary growth step under hydrothermal conditions. Contrary to this description, we used in this paper an in situ method with OSDA to form the seeding layer directly on a porous stainless steel support, followed by a calcination step to remove the OSDA. In a further step, an OSDA-free layer of zeolite beta was generated on top of the seeded, but nearly organic free support structure by a seed-induced crystallisation. A more detailed description was published earlier [34]. Additionally, the progress of this method was characterized stepwise mainly by X-ray diffraction (XRD) and scanning electron microscopy (SEM) measurements and gas separation experiments to prove the idea of the preparations sequence and their ability to act as membranes, respectively.

## 2. Results

### 2.1. Characterization of the OSDA-Free Zeolite Beta Layer

Porous stainless steel support structures with an intermediate TiO_2_ layer were seeded via a multiple step procedure: several times with an OSDA containing, zeolite beta synthesis procedure by multiple in situ crystallization (MISC) method, followed by high-temperature calcination and an OSDA-free secondary growth method, in order to create an OSDA-free layer. In Table 1, an overview of the prepared membranes with different numbers of seeding steps with OSDA followed by one or two secondary growth steps without an OSDA is given. An inductively coupled plasma optical emission spectroscopy (ICP-OES) analysis of the resulting excess powder revealed a Si/Al ratio of about 17 for the zeolite beta seed crystals. It is assumed that the seed layer has the same ratio. The OSDA-free powder, prepared under similar conditions as the layer, was used for ICP and has a Si/Al ratio of about 4.5.

**Figure 1:**
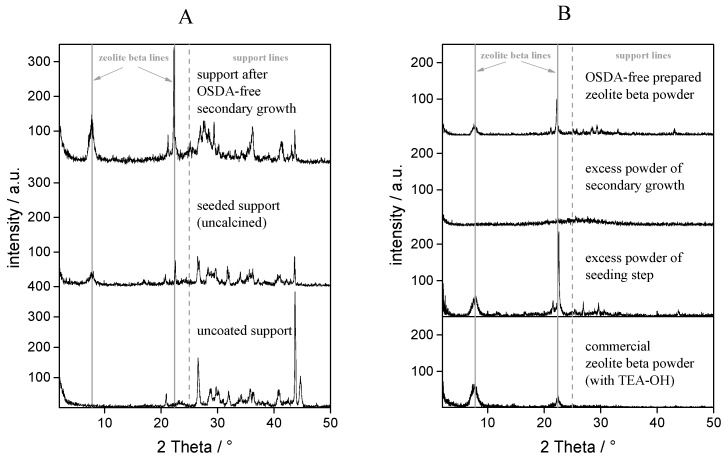
XRD patterns of ‘uncoated support’ (TiO_2_ covered stainless steel), ‘seeded support’ (in situ seeded: membrane number 2.1) and ’OSDA-free secondary growth’ (membrane number 2.2) for experimental series 2 (**A**). In addition, the related excess powders as well as a beta powder prepared without an OSDA and commercial zeolite beta powder prepared with TEAOH for comparison (**B**).

**Figure 2:**
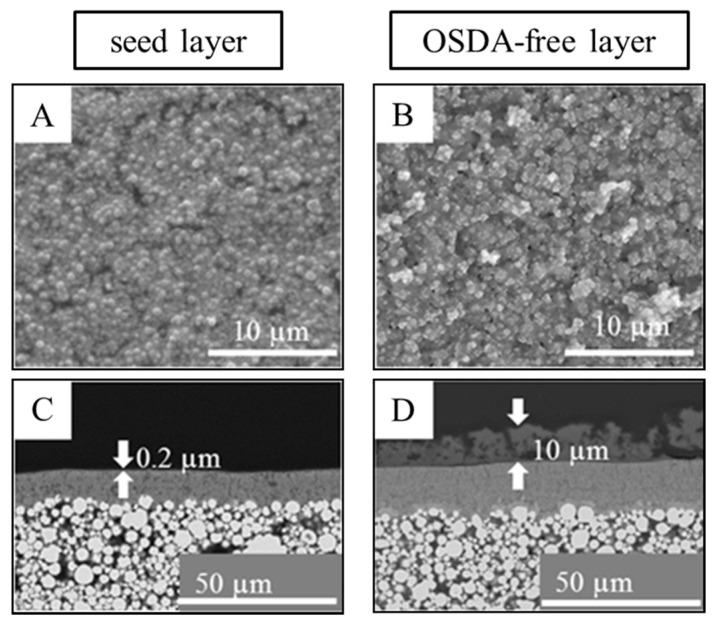
SEM (top view and cross section) of the experimental series 3: layer after the seeding step (membrane number 3.1—(**A**,**C**)), and after the OSDA-free secondary growth step (membrane number 3.2—(**B**,**D**)). The overall layer thickness of about 10 µm is a zeolite beta layer, which is divided into two sections: a very thin, denser zeolite beta base layer of about 1 µm (not marked) and a porous and loosely packed, thicker part.

The XRD analysis presented in Figure 1A shows the pure support, the seed layer and the OSDA-free layer after a secondary growth step, compared to a diffraction pattern of a commercial zeolite beta powder prepared with tetraethylammonium hydroxide (TEAOH) and the resulting excess powders produced in the bulk phase during the hydrothermal synthesis step and the secondary growth step (Figure 1B). These XRD patterns indicate clearly that zeolite beta is obtained on the top of the support structure after a seed-induced synthesis method, and without the addition of further OSDA. The change in the diffraction pattern in the 2θ range bet 25° and 35° of the uncalcined seeded support and the OSDA-free secondary growth process is due to the transformation during the calcination at higher temperatures of the TiO_2_ coating from a mixed anatase/rutile phase to a rutile only phase (Figure 2a). After seeding, a very thin layer is formed, and, therefore, the peaks are less intense, but the excess powder shows the formation of zeolite beta. The higher intensity of the zeolite beta peaks indicates that the amount of zeolite beta can be increased by the OSDA-free method, which can be proved as well by a weight increase in the composite structures. After one seeding step, about 12 mg × cm^−2^ zeolite was distributed over the support structure; the zeolite amount was increased to about 23 mg × cm^−2^ after the secondary growth step.

SEM analysis (Figure 2) shows a homogenously distributed zeolite beta layer after the seeding step with a layer thickness of about 0.2 µm. After the OSDA-free secondary growth step, the crystal size slightly increased but remained as a well-distributed layer. Cross section analysis shows an overall layer thickness of about 10 µm, which is divided into two sections: a very thin, denser zeolite beta base layer of about 1 µm and a porous and loosely packed, thicker part. 

The diffraction pattern of the excess powder can be seen in Figure 1B. If TEAOH is used as OSDA in the bulk phase, zeolite beta powder is produced in parallel to the seed layer that is formed on the support material. In the second step, we assume that the seed crystals are securely attached to the support and will not be distributed into the surrounding synthesis solution. This conclusion is supported by the structure of the excess powder from the OSDA-free synthesis experiment, which is completely amorphous (see Figure 1B). This means no seeding occurs in the solution itself.

**Figure 3:**
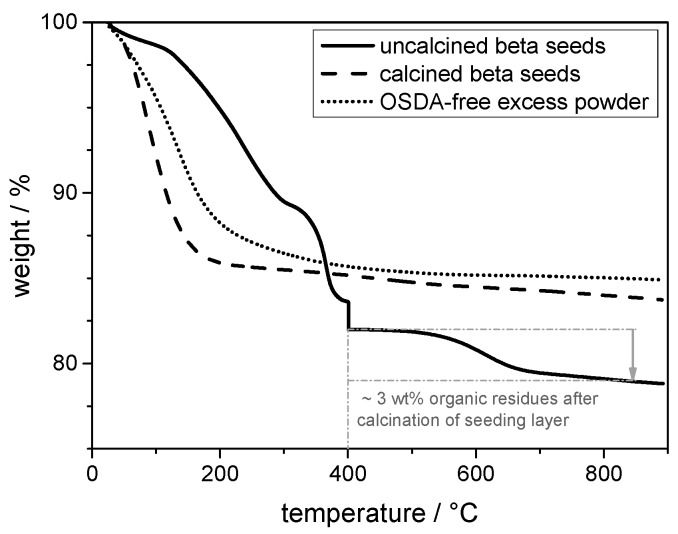
TGA curves of beta seeds calcined at 550 °C (prepared with OSDA), the OSDA-free excess powder formed during the secondary growth step, and simulated calcination process at 400 °C of non-calcined beta seeds as it was used for the calcination of the seed layers.

The seeded support structures should not be calcined above 400 °C because of the temperature stability of the porous TiO_2_ coated stainless steel, so approximately 3 wt% of the total weight, which means approximately 30% of the decomposed organic material, is left inside the seed crystal (straight-line). Nevertheless, we were able to show that these residues do not influence the secondary growth process itself. Thermogravimetric analysis (TGA) curves (Figure 3) prove this fact. In the TGA curve of the OSDA-free excess powder (dotted line), no organic decomposition steps are visible indicating that the excess powder does not contain any decomposable organic material. Consequentially, the seeded support does not release any organic material, which might affect the crystallisation process. 

**Figure 4:**
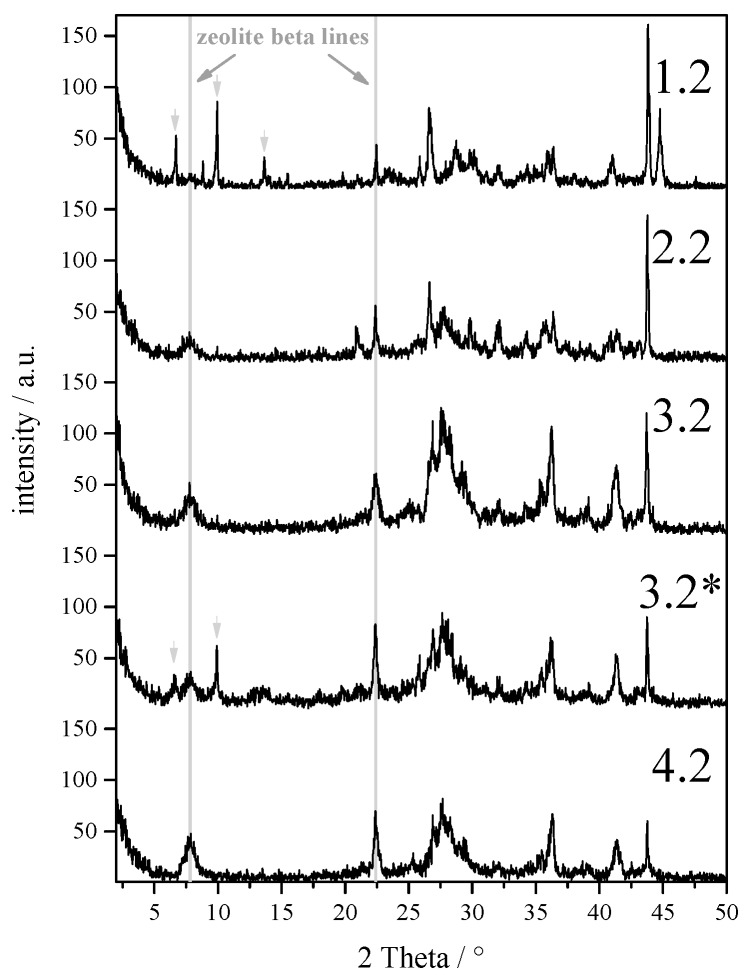
Diffraction patterns of the prepared membranes after the OSDA-free secondary growth step. On series 3.2*, two consecutive OSDA-free secondary growth steps were performed. Impurities of mordenite are marked in the diffraction pattern with an arrow.

**Figure 5:**
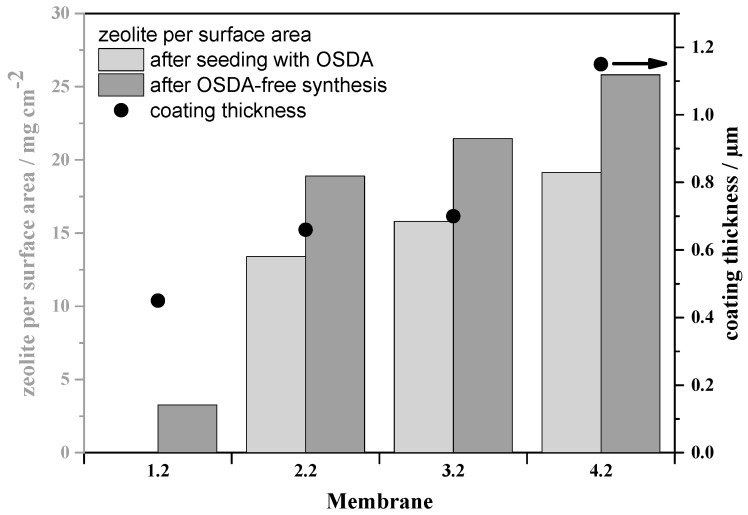
Zeolite mass per surface area (columns) and thickness of the resulting beta layer (dots) on the porous support after the OSDA-free secondary growth step (the membranes 1.2 to 4.2 representing the variation of the number of seeding procedures (see Table 1)).

The support structures were seeded several times with an OSDA containing zeolite beta by a MISC method, in order to evaluate the influence of the seeding amount on the quality of the resulting membrane. In Figure 4, the diffraction patterns of the synthesized membranes with different seeding steps and secondary growth steps are given. It is definitely realized that an increasing number of seeding steps shows higher intensity for the characteristically zeolite beta peaks. This goes along with the amount of zeolite on the membrane and the thickness of the resulting zeolite beta layers. In Figure 5, it is shown that an increasing number of seeding steps result in a thicker zeolite beta layer and a higher mass per surface area of zeolite beta. Consequently, the thickness of a membrane with three seeding steps followed by a secondary growth procedure has a higher thickness. The execution of the secondary growth step after two seeding steps, membrane 3* shows no improvement of the zeolite phase and its amount, Figure 4. The XRD diffraction patterns further show impurities for the membranes 1.2, and 3.2*, which are belonging to the mordenite structure, as it was also formed during the OSDA-free secondary growth process.

### 2.2. Membrane Preparation with OSDA-Free Beta

**Figure 6:**
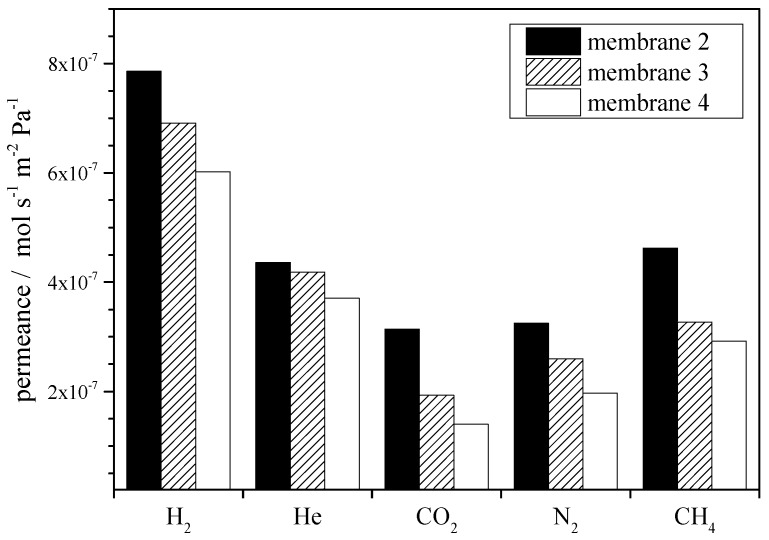
Comparison of single gas permeance of small gases for the differently prepared membranes (2.2, 3.2 to 4.2 with increasing numbers of seeding steps 1, 2 to 3 numbers, respectively (see Table 1).

Zeolite beta coated materials are applied as structured catalytic reactors and sensors [6,7,8]. Furthermore, the application as membrane has garnered increasing interest in recent years; we also evaluated the quality of the coated layers using single gas permeance measurements. The permeances of the gases He, H_2_, CO_2_, N_2_ and CH_4_, measured at room temperature with a pressure difference between feed and permeate side of about 1 bar for the membranes 2.2, 3.2 and 4.2, are depicted in Figure 6. Thereby, the permeance of molecules with smaller kinetic diameter is higher than the permeance of bigger molecule. In addition, it is clearly shown that, with an increasing number of seeding steps, the permeance decreases. 

**Figure 7:**
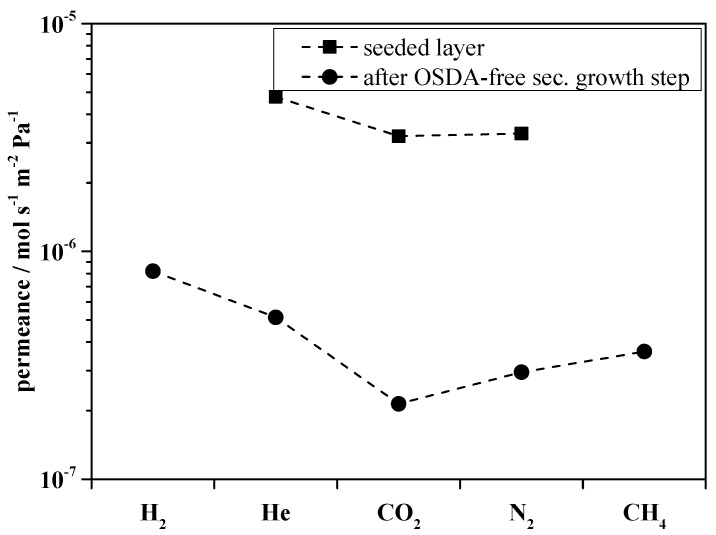
Comparison of the single gas permeance of small gases for the in situ seeded membrane 2.1 (square) and the OSDA-free layer membrane 2.2 (circle).

After the seeding step and calcination, high permeances were measured. These were reduced by more than one order of magnitude after the secondary growth step, which is shown in Figure 7. The reproducibility is still low if only one seeding layer is applied. The coverage of the surface was very low with only one seeding step so that two seeding steps were tested. The amount of zeolite beta was increased and the surface looked more homogeneous than after only one seeding step. The thickness of the layer was around 0.6 µm and the affinity to crack was higher. After the second seeding, the permeances for the single gases decrease and the selectivity for CO_2_ over N_2_ is slightly improved.

## 3. Materials and Methods 

### 3.1. Preparation of the Zeolite Beta Seed Layer on Porous Stainless Steel Supports

The zeolite beta seed layers were prepared from a molar gel with a composition of 1 SiO_2_:0.56 TEAOH:0.02 Al_2_O_3_:15 H_2_O. Ludox AS40 (40%, Sigma Aldrich, St. Louis, MO, USA) and Al(NO_3_)_3_·9 H_2_O (98%, Fluka, St. Louis, MO, USA) were used as silica and alumina sources, respectively. Tetraethylammonium hydroxide (TEAOH, 40% in H_2_O, Sigma Aldrich) was used as OSDA. The silica source and the OSDA were stirred for 1 h before the alumina source, dissolved in distilled H_2_O, was added drop-wise and the mixture was stirred for another hour. As support, stainless steel discs covered with an additional, intermediate TiO_2_ layer, provided by GKN Sinter Metals Filters GmbH (Radevormwald, Germany) (SIKA-R 0.1 TiO_2_, 18 × 2 mm), of 18 mm diameter were used. The support structure and the synthesis mixture were transferred together into a Teflon-lined autoclave. The support was placed in a Teflon holder in a slightly vertical position with the fine side facing up. The detailed procedure has been described elsewhere [17,35]. The hydrothermal synthesis was carried out at 150 °C for 48 h. This procedure was repeated several times to increase the amount of seed crystals and to gain a more homogenous distribution on the support material via these multiple in situ crystallization steps. The resulting excess powder was centrifuged, washed with distilled H_2_O and dried at 75 °C. The resulting excess powder, produced in the bulk phase, was analysed by TGA and ICP-OES, assuming that it had the same composition and properties as the zeolite layer. The seeded supports were calcined at 400 °C with a heating rate of 0.2 °C min^−1^ and kept at isothermal conditions for 16 h to remove the OSDA. During the calcination step, synthetic air, with a flow rate of 45 mL × min^−1^, was used. The excess powder was calcined at 550 °C with a heating rate of 3 °C × min^−1^ for 6 h.

### 3.2. Synthesis of OSDA-Free Zeolite Beta Layer

The OSDA-free zeolite beta layer was prepared from a molar gel composition of 1 SiO_2_:0.36 Na_2_O:0.025 Al_2_O_3_:40 H_2_O, [27], using fumed silica (99.8%, Sigma Aldrich) and NaAlO_2_ (Al_2_O_3_ 50–56%, Na_2_O 40–45% Riedl-de-Haen, Seelze, Germany) as silica and alumina sources, respectively. NaOH (97% Merck, Darmstadt, Germany) was used as mineralizing agent. Distilled water, NaAlO_2_ and NaOH were stirred for 1 h before the fumed silica was added slowly to the mixture, which was stirred again for 2 h more. The seeded support and the synthesis mixture were transferred together into a Teflon-lined autoclave. The support was again placed in a slightly vertical position. The hydrothermal synthesis was carried out at 140 °C for 72 h. For preparing OSDA-free powder, 11.9 wt% based on the SiO_2_ amount, of calcined excess powder, formed during the first synthesis step, was suspended in the synthesis mixture as seed crystals.

### 3.3. Characterization and Single Gas Permeances

The zeolite beta coated supports and the excess powders were characterized by XRD by means of an X’Pert Pro diffractometer (Philips Analytical, Almelo, The Netherlands) with Cu-Kα radiation. SEM of the top view and cross section of the layers was carried out with a FEI Quanta 200 (FEI Company, Hillsboro, OR, USA). For the cross sectional images, the membrane was placed in an epoxy resin and cut with a diamond saw, followed by polishing and sputtering with gold. TG measurements of the excess powders were performed with the SDT 2960 (TA Instruments, New Castle, DE, USA). The measurement was made with a heating rate of 10 °C × min^−1^ from room temperature until 900 °C under an air flow of 100 NmL × min^−1^. The Si/Al ratio of the excess powder was determined by ICP-OES Ciros CCD (Spectro, Kleve, Germany). 

The quality of the zeolite coatings was evaluated by single gas permeance measurements. The disc-shaped membrane was placed in a stainless steel cell and sealed with an O-ring made out of viton. An electrical heating system was used to heat up the membrane cell and the pipes. For the single gas measurements, He, H_2_, CO_2_, N_2_ and CH_4_ were dosed into the set-up with a flow rate of 100 mL × min^−1^ by mass flow controllers. The pressure at the feed side was varied between 1 and 2 bars. The permeate side was kept at the atmospheric pressure. The resulting permeate flow was measured with bubble flow meters of varying sizes. In a first step, the membranes were heated up in situ to 200 °C in N_2_ atmosphere. This temperature was hold for 6 h to remove any adsorbed or trapped moisture. The measurements for He, CO_2_, N_2_, H_2_ and CH_4_ were then carried out at room temperature. 

## 4. Conclusions

The preparation of OSDA-free, thin and active zeolite layer is of great importance e.g., for sensing, separation and catalytic applications, in particular for the preparation of zeolitic membranes to avoid a calcination step at higher temperatures of the whole device finally. Such a thermal treatment would cause stress, due to the different expansion coefficients of the support and zeolites, and, as a consequence, defects in the membrane itself. With this paper, we present a new systematic approach for the preparation of OSDA-free zeolite beta layers. We are going to use especially porous stainless steel supports—covered with a TiO_2_ intermediate layer—on which a zeolite beta layer will be formed in an OSDA-free synthesis route. Such syntheses are reported mainly for powders. We adapted and modified such a preparation route for zeolite beta layers and characterized the resulting membrane like system. Therefore, we propose in this paper a two-stage process, which includes an in situ seeding (step 1—eventually multiple seeding) followed by a secondary growth process (step 2). 

In particular, we prepared different membranes with a different number of in situ seeding steps to increase the seed amount systematically. The following secondary growth step was carried out only once. For comparison, an experimental series has been carried out with a second consecutive secondary growth synthesis. As expected, the amount of zeolite beta, seeded on the support, increases with additional in situ seeding steps. The thickness of the final OSDA-free zeolite layer is increasing systematically with number of seeding steps by just one secondary growth step, where no OSDA is used. However, the layer prepared without an OSDA is thicker than for membranes prepared with OSDA. Thus, a final calcination step at the end of the preparation procedure can be avoided.

In the application tests, single gas permeation measurements have been carried out with H_2_, He, CO_2_, N_2_ and CH_4_. Two main conclusions can be drawn: (i) the high permeance of small single gases after the seeding step could be reduced by more than one order of magnitude by the OSDA-free secondary growth step, which documents the success of this two-step procedure—a denser zeolite beta layer could be prepared; (ii) in addition, this effect could be improved by increasing the number of in situ seeding steps if, finally, an OSDA-free secondary growth step follows.

Hence, additional optimization of the preparation route is on the way. Such experiments include a systematic repetition of the OSDA-free secondary growth step and the use of an ex situ seeding step, in order to realize a complete OSDA-free membrane preparation procedure. In addition to the synthesis optimization already presented here, a systematic variation of the Si/Al ratios in the zeolitic layers is ongoing to adjust the ion-exchange capability of the zeolites in the device. With this, two modifications can be investigated: (i) the acidity of the zeolite induced by an ammonia exchange and an additional mild thermal treatment and (ii) the pore design via using different kinds of cations and/or cation exchange levels.

## 5. Patents

The following patent results from the reported work: S. Reuss; W. Schwieger; M. Schülein; B. Reif; S. Basahel; A. Al-Youbi; S. Al-Thabaiti. Process for the preparation of organo-template free supported zeolite layers, Germany, 2015, WO 2015001095 A1.

## Figures and Tables

**Table 1:** Overview of the prepared membranes with increasing numbers of seeding steps with OSDA followed by one secondary growth step without OSDA. For the marked (*) sample, two consecutive secondary growth steps were performed.

Experimental Series	Seeding Steps (with OSDA)	Membrane Number	Secondary Growth (without OSDA)	Membrane Number
1	0	1.1	1	1.2
2	1	2.1	1	2.2
3	2	3.1	1	3.2
3 *	2	3.1 *	2	3.2 *
4	3	4.1	1	4.2
